# Dengue Virus Infection: A Tale of Viral Exploitations and Host Responses

**DOI:** 10.3390/v13101967

**Published:** 2021-09-30

**Authors:** Nikita Nanaware, Anwesha Banerjee, Satarupa Mullick Bagchi, Parikshit Bagchi, Anupam Mukherjee

**Affiliations:** 1 Division of Virology, ICMR-National AIDS Research Institute, Pune 411026, MH, India; nikitananaware20@gmail.com (N.N.); banerjee.anwesha1991@gmail.com (A.B.); 2 Life Sciences Institute, University of Michigan, Ann Arbor, MI 48109, USA; smbagchi@umich.edu; 3 Department of Cell and Developmental Biology, University of Michigan Medical School, Ann Arbor, MI 48109, USA

**Keywords:** Dengue virus, viral pathogenesis, Dengue genome, viral replication, inflammation, host immune response, cytokine storm, Antibody Dependent Enhancement (ADE), apoptosis, autophagy

## Abstract

Dengue is a mosquito-borne viral disease (arboviral) caused by the Dengue virus. It is one of the prominent public health problems in tropical and subtropical regions with no effective vaccines. Every year around 400 million people get infected by the Dengue virus, with a mortality rate of about 20% among the patients with severe dengue. The Dengue virus belongs to the Flaviviridae family, and it is an enveloped virus with positive-sense single-stranded RNA as the genetic material. Studies of the infection cycle of this virus revealed potential host targets important for the virus replication cycle. Here in this review article, we will be discussing different stages of the Dengue virus infection cycle inside mammalian host cells and how host proteins are exploited by the virus in the course of infection as well as how the host counteracts the virus by eliciting different antiviral responses.

## 1. Introduction

Dengue is a single-stranded positive-sense RNA virus, a member of the Flaviviridae family that causes dengue fever. This virus spreads through the bite of infected Aedes mosquitos [1]. Dengue fever is a major public health threat worldwide. It is affecting about 3 billion people, which are forty percent of the world population residing across more than 100 nations around the world, wherein tropical and subtropical regions are the worst affected, whereas 100–400 million people are infected each year. Dengue incidence has increased more than eight-fold over the past 20 years [1]. Weak mosquito control policies, afforestation, climate change, and global warming are few of the reasons for this increase. Severe Dengue virus infection causes tenderness and belly pain, vomiting at least thrice a day, epistaxis, hematemesis, melena, fatigue, and restlessness, which finally lead to hemorrhagic fever and organ failure [2]. Four different serotypes of the Dengue virus are found worldwide. They can cause a mild self-limiting infection or severe Dengue hemorrhagic fever (DHF) or Dengue shock syndrome (DSS), which leads to approximately 20,000 fatalities annually. Conventional vaccines are in progression, but the co-circulation of different serotypes and antibody dependent enhancement events constrict vaccine development [3]. Due to these reasons, Dengue virus (DENV) continues to persist as one of the most interesting viruses in the field of infection biology.

The Dengue virus has four popularly known serotypes, namely, DENV-1, DENV-2, DENV-3, and DENV-4, with the recent discovery of a fifth serotype in the year 2013 [4]. Although the serotypes of DENV share around 65% similarities, an infection with different serotypes manifests a range of clinical symptoms.

Besides viral exploitations of the host cellular machinery, a host cell also exerts various antiviral responses. The host immune system acts in multiple ways to combat against the virus infection such as the induction of inflammation, interferon dependent cytokine production, and cell death by triggering apoptosis or autophagy. In this review, we discuss steps crucial in the Dengue virus replication cycle in mammalian cells, its pathogenicity, as well as different antiviral responses exerted by the host cell. Knowledge of the Dengue virus replication cycle and information about host proteins exploited by the virus can be very important to develop antiviral targets and have immense importance in public health.

## 2. Structural Details of Dengue Virus

**Figure 1:**
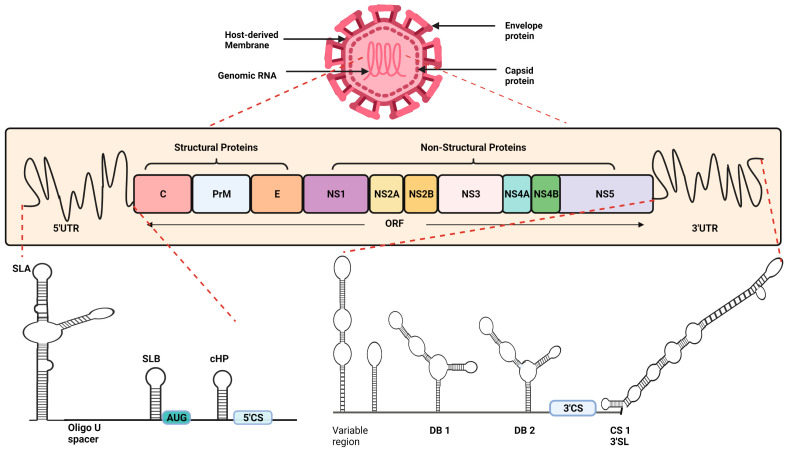
**Structural organization of Dengue virus genome:** The viral genome consists of 5′UTR, an ORF, and 3′UTR. The ORF encoding polyprotein serves as a template for the translation of 3 structural proteins (C (Capsid), PrM (Pre-Membrane), and E (Envelope)) and 7 Non-structural proteins (NS1, NS2A, NS2B, NS3, NS4A, NS4B, NS5). The representation of 5′UTR depicts Stem-loop structures (SLA and SLB) flanked by oligo U, wherein SLB is followed by initiator AUG and the hairpin structure (cHP) in the C encoding region. The following complementary sequence at 5′UTR is essential for genome circularization, which in turn is important for genome replication. The 3′UTR consists of variable sequences, followed by dumbbell structures DB1 and DB2. The approaching conserved sequence CS1 and stem loop (SL) at 3′UTR play a pivotal role in genome conformational changes facilitating replication.

The Dengue virus, roughly spherical in shape, is an enveloped single-stranded positive-sense RNA virus with an icosahedral nucleocapsid covered by the lipid bilayer. The 11 kb long DENV genome can function as mRNA, and, similar to that in eukaryotes, there are untranslated regions (UTRs) at both the 5′ and 3′ end flanking the open reading frame (ORF) (Figure 1). The type I 5′ cap serves as an initiation site for translation, while the 3′ end lacks the poly-A tail and instead has a stem-loop. Within the 5′UTR, there are stem-loop (SL) structures—SLA and SLB, upstream and downstream regions concerning AUG denoted as the 5′UAR (upstream AUG region) and 5‘DAR (downstream AUG region), C-coding hairpin (cHP), and 5′ cyclization sequences (5′CS). The DAR, cHP, and 5′CS are located in the C protein encoding region. The 5′UTR comprises sequences of 95–101 nucleotides in the four DENV serotypes, DENV-1 to DENV-4, while the 3′UTRs’ length is variable among the serotypes [5].

The ORF flanking by UTRs encodes a polyprotein, which is the precursor to 10 mature proteins. Processing of this polyprotein both co- and post-translationally contributes three structural and seven non-structural proteins. As the name suggests, structural proteins—Capsid (C), Envelope (E), pre-Membrane (prM)—are the structural component of a virus particle, involved in the encapsidation of viral RNA, i.e., nucleocapsid formation, membrane formation, maturation, and envelopment of the virion. The non-structural proteins NS1, NS2A, NS2B, NS3, NS4A, NS4B, and NS5 have diverse enzymatic activities that are being explored for their multiple roles in an infectious cycle [5,6].

To highlight a few roles of NS proteins: NS5 is viral RNA-dependent RNA polymerase (RdRp), and possesses nuclear localization sequences (NLS) as well as *S*-adenosyl methionine methyltransferase (MTase) activity; NS3 has RNA-triphosphatase (RTP), nucleoside triphosphatase (NTPase), and helices activities, providing abilities on both NS3 and NS5 to contribute vitally to RNA replication. NS2B is a viral serine protease and, along with other NS proteins NS1, NS2A, NS4, and NS4B, plays diverse roles in viral replication, assembly, and release [5,6].

## 3. Transmission and Replication Cycle of Dengue Virus inside Mammalian Cell

The Dengue virus is transmitted by female mosquitoes, mainly of the species *Aedes aegypti*, and to a lesser extent by a few other species such as *Aedes albopictus, Aedes polynesiensis, Aedes scutellaris*, etc. The virus is transmitted to humans from an infected mosquito during the ingestion of blood. It is essential for an RNA virus such as DENV to establish contact, bind, and penetrate the susceptible host to gain access to host cellular machinery for its own multiplication. The Dengue virus is released from the salivary gland of the pathogenic female *Aedes aegypti* mosquito via saliva into the skin of the mammalian host. Viral replication starts in the secondary tissue of the vector, such as the salivary glands [7]. This replication leads to virion release in the saliva prior to the virus transmission to the human host.

DENV binds to a variety of molecules, widening its horizon for infecting diverse cell populations that include but are not restricted to epithelial cells, fibroblast, monocytes, macrophages, dendritic cells, B cells, T cells, endothelial cells, and hepatocytes [8]. A detailed account of the events initiating at viral attachment until release is made in the following sub-sections.

### 3.1. Entry of Virus Particle and Role of Cell Surface Receptors

**Figure 2:**
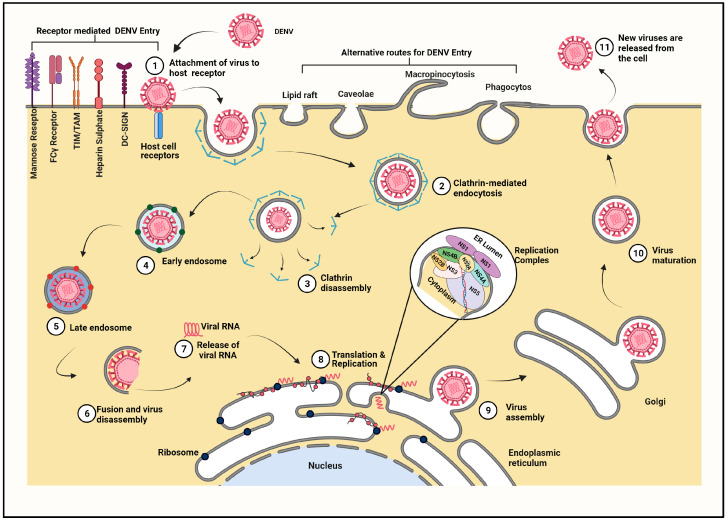
**Replication cycle of Dengue virus:** The replication cycle begins with entry into the host cell that initiates from binding of the virus to host cell receptors. (1) The DENV bind can host various receptors to enter via receptor-mediated endocytosis, while alternative routes of entry are described too. Most studied and reported, clathrin-mediated endocytosis is described hereafter. (2) The clathrin-coated pit serves as the site of internalization, and the virus is endocytosed into a perforated bubble coated with clathrin. (3) Further, the clathrin is released, initiating endosomal processing. (4) The early endosome is marked by presence of the Rab5 protein (green solid spheres) where pH is around 6.5, which proceeds towards endosome maturation. (5) The mature endosome is marked by Rab7 (red solid spheres) and at a lower pH, i.e., around 5.5, this acidic pH leads to various conformational changes. (6) The conformational changes cause fusion of the viral envelope and host membrane. (7) The disassembly of the virus results in the release of the encapsidated viral genome (capsid-bound RNA) into the cytoplasm. (The mechanism of uncoating the viral genome is unclear and thus not described in the figure.) (8) The viral RNA serves as a template for translation and replication in the ER. The special sight for replication, formed by convolution of the ER membrane in the presence of NS proteins is called the Replication complex. (9) The replicated genome and translated viral proteins are assembled, forming an immature virus particle in the ER. (10) This immature virus undergoes furin-mediated maturation in the Trans-Golgi Network (TGN). (11) The mature virus is then exocytosed from the infected cell, completing the infection cycle.

Various attachment-internalization assays, inhibition studies, mutational experiments, electron micrographic studies, live-cell imaging, and virus trafficking studies helped to identify various attachment factors that facilitate the surface binding and cellular receptors involved in the internalization of the virus particle (Figure 2). The numerous host receptors known to be associated with the viral entry—including, but not restricted to, the mannose receptor (MR) reported in monocytes, macrophages, and the mouse embryonic fibroblast cell line 3T3; heparan sulfate, a type of glycosaminoglycan (GAG) which acts as the receptor in epithelial cell lines such as Vero and CHO K1; the lipopolysaccharide (LPS)-CD14 complex—were suspected to be receptors of immune cells, such as the monocyte and macrophages, and were elucidated when the role of heat-shock proteins, HSP70, and HSP90 in DENV entry was revealed [9]. A c-type lectin, known as Dendritic cell-intercellular adhesion molecule 3-grabbing nonintegrin (DC-SIGN), helps the virus to invade dendritic cells. Besides these majorly studied cells and receptors, DENV infects human hepatocyte cells via a chaperonin named GRP78, while the AXL protein grants entry of DENV into human primary astrocytes and epithelial cells (A549, Vero, human primary epithelial cells). Not only AXL but also Phosphatidylserine (PS) receptors, T cell Ig mucin (TIM), and TYRO3, AXL, or MERTK (TAM) family receptors, are thought to be serving as prime receptors or co-receptors of DENV entry. Besides that, various other molecules were also studied for their contributions in DENV entry, to name a few: glycosphingolipids are studied in K562, BHK-21, the high-affinity lamini receptor in HepG2 cells, and claudin in Huh-7 and Huh-7.5 cells. Additionally, a few more molecules (proteins reported as 65kDa, 44kDa, 74kDa, etc.) were investigated for the same reason [8,9,10]. Above and beyond, Fcγ receptors are known to be facilitating an interesting phenomenon, wherein heterologous secondary infection in the presence of sub-neutralizing antibodies enhances DENV infection, known as Antibody Dependent Enhancement [10].

The viral counterpart interacting with these host cell surface receptors and co-receptors is the surface protein, Envelope (E). The E protein is a glycoprotein that has three domains, the C-terminal transmembrane (Immunoglobulin constant) IgC similar to the anchor domain that recognizes and binds to the host cell, the central domain-linking anchor, and ectodomain that is involved in attachment and internalization of the virus. While the viral E protein actively participates, the Membrane protein of the virus is reported to be facilitating the viral binding to the host cell receptor. The Membrane protein in a mature virus or Precursor-Membrane (prM) protein in immature virus particles comprises the N-terminal pr-domain that prevents premature virus-membrane interactions, followed by the M-domain that forms the membrane of the mature virus, and a stem region that interacts with the E-protein, aiding in the maturation of the virion. The pr peptide present in the immature virion is cleaved off by furin, leading to maturation of virus particles [5,6,11].

The binding of the virus to the host cell receptor is crucial in establishing infection with most reports on clathrin-dependent receptor-mediated endocytosis for DENV entry. However, clathrin-independent receptor-mediated endocytic entry is also reported. There are a few reports of alternative routes of entry such as direct fusion or diffusion, micropinocytosis, etc. [9]. Differences in the tropism or infectivity of the virus depend on the serotype of the infecting strain and also on its emergence, i.e., the host cell in which the virus propagated.

There are several studies that describe the origin-based tropism, wherein it was observed that DC-derived viruses could infect only cells expressing L-SIGN but not the DC-SIGN expressing one, while the virus propagated in other cells could infect both DC-SIGN and L-SIGN expressing cells. This differential infection pattern was explained by the observation that DC-derived DENV lacked mannose-rich glycan that is important for establishing infection in DC [9].

### 3.2. Fusion and Release of Viral Genome

After binding to the cognate receptors of host cells, the virus is internalized into a clathrin-coated bubble-like structure, known as the endosome (Figure 2). The virion is majorly found to be internalized into the early endosome having Rab5 and later either matures into or fuses with the pre-existing late endosome having Rab7 [12]. When the internalized virus experiences an acidic pH inside the endosome, the E protein (dimer) and PrM-E (trimer) undergo various conformational changes. However, comparatively, the PrM containing the immature virus has to endure a few additional changes [13]. In mature viruses, the dissociation of E protein homodimers, where domain II of the E protein gets hinged outward from the virus surface, leaves the fusion loop exposed, which is followed by a rearrangement of the E protein. The fusion loop at the tip of Domain II interacts with the host endosomal membrane leading to trimerization of the E protein. The trimerization spans from the tip to the base which causes conformational changes such as the rotation of Domain III, shifts, and the displacement of trimers leading to the fusion of the viral envelope and the host’s endosomal membrane. The presence of anionic lipids in the late endosomes plays an important role in viral as well as endosomal membrane fusion leading to viral nucleocapsid deployment in the cytosol [14].

Few alternative virus trafficking pathways are reported that include the clathrin-independent receptor-mediated endocytosis, direct penetration through the plasma membrane of the host cell through fusion, or diffusion, and are known to release the nucleocapsid into the host cytoplasm [9].

### 3.3. Transport of Viral Genome

The highly basic capsid protein binds the viral RNA with great affinity but little specificity. The C protein uncoating the viral genome in the nucleocapsid is lost via an unknown mechanism; however, the non-degradative step of ubiquitination is suspected to play a role in genome uncoating. The recent studies stated that inhibition of ubiquitination blocked the DENV genome uncoating wherein ubiquitin E1-activating enzyme inhibition stabilized the viral genome by retaining it into the endosomes or nucleocapsids during infection [5,15]. Hereafter, the cytoskeletal machinery that undergoes constant modification is thought to be facilitating the viral genome translocation of the uncoated viral genome to the rough endoplasmic reticulum (RER); however, the exact mechanism is not known yet [16]. This transported positive sense viral RNA can directly act as mRNA for protein synthesis as well as act as a template strand for viral genome replication.

### 3.4. Viral Replication

The viral genome transported to the endoplasmic reticulum (ER) serves the purposes of synthesizing viral proteins and replicating the viral genome (Figure 2).

The replication of the viral genome is coupled to the translation of the polypeptide, as viral proteins are prerequisites for RNA replication. An ER resident multi-subunit protein complex, named the ER membrane protein complex or EMC, plays a very important role in the biosynthesis of the Dengue virus polyprotein [17,18,19]. DENV replicates with the help of a replication complex, which is a cytoplasmic compartment that protrudes into the ER [20]. The same positive-sense RNA strand can serve as a template for both synthesizing proteins as well as the complimentary negative strand [21]. The viral genome structure facilitates both the processes; the linear form is dedicated to translation and protein synthesis, while the circular form is devoted to transcription [20]. The C-terminal end of the NS3 protein has three enzymatic properties: a 5′ RNA-triphosphatase (RTP), a nucleoside triphosphatase (NTPase), and a helicase. NS3 forms a complex with NS5 and assists in replication through the unwinding of viral RNA and dephosphorylation prior to 5′-end capping [22].

The translation and maturation of viral proteins lead to conformational changes in the membranes of the ER to form convoluted membranes and vesicle packets that are connected to the cytoplasm via pore-like structure for supplying the essential factors such as nucleotides [23]. The vesicle packets contain NS5 (RdRp), NS3 (possessing enzymatic properties: helicase), 5′ RNA-triphosphatase (RTP), a nucleoside triphosphatase (NTPase), and other viral NS proteins in addition to the host factors that catalyze RNA replication, collectively known as the replication complex [11]. The SLA at 5′UTR acts as a promoter for NS5, the viral RdRp [24]. The relocation of NS5 to the 3′end is facilitated by cyclization of the genome for the initiation of RNA synthesis. The base pairing between the complimentary sequences of 5′ and 3′ UAR unwraps 3′ SL making it available for replication [22]. The priming required for RNA elongation is carried out by NS5 via *de novo* synthesis of a CU dinucleotide [25]. The newly synthesized negative stand remains bound to the template positive stand forming dsRNA [26]. Further, this negative strand serves as a template, and the newly synthesized nascent positive strand displaces the pre-existing one and forms dsRNA intermediate. This dsRNA intermediate amplifies positive strands of viral RNA. Thus, RNA replication is asymmetric, generating a 10-fold greater number of copies of positive-stranded RNA than negative-stranded RNA [6]. Capping and methylation of the positive-stranded RNA occur co-transcriptionally, observed in genomic viral RNA but not absent in dsRNA intermediates [27]. These new positive sense ssRNAs can be either translated further or encapcidated to facilitate raising new viral particles.

### 3.5. Assembly of Viral Proteins

Encapsidation of the viral genome, also termed as nucleocapsid (NC) formation, is an electrostatic interaction resulting in the binding of the positively charged capsid protein to the negatively charged RNA [28]. The newly synthesized viral positive sense RNA binds to NS2 via interactions between 3′UTR of RNA and the R94, K95, and K99 residues of NS2 [29]. NS2 is also observed to recruit the translated C-prM-E polyproteins along with NS2B-3 proteases to the assembly site [15,29,30,31]. The C and prM proteins are linked with an anchor (a hydrophobic signal peptide), which spans the ER membrane. The C-anchor junction is cleaved by NS3B in the presence of NS2B as a cofactor collectively called NS2B-3, acting as serine protease, which leads to free prM and a mature capsid protein for binding with the RNA, thus forming the nucleocapsid [28,32]. The NC buds along with the ER membrane in the presence of E and prM proteins into the ER lumen and travels through the secretory pathway as an immature virus particle wherein the pr domain of prM caps the fusion loop of the E protein to avoid premature fusing of the immature virion to the host membrane [32].

### 3.6. Maturation and Egress of Virus

The E protein of the partially assembled immature virion experiences reversible pH-dependent conformational changes following the secretory pathway during the egress of virions. Trimers of prM-E heterodimers are the part of immature virus particles, while a mature virus consists of homodimers of the E protein and is observed upon fusion of the viral and host endosomal membrane [33]. The virus particle travels through the Golgi into the TGN where the prM protein is cleaved by the protease furin, causing a transition of the spiky immature virus particle into a smooth mature particle [13,34,35]. However, the continued association of the pr segment with the virion is observed until it is released from the host cell. This retention of the pr segment during transit of the virus in the acidic environment TGN prevents premature binding of the E protein to the exosomal membrane [36]. In addition to that retention, pr could be needed for other viral activities such as an interaction with the vacuolar-ATPases (V-ATPases) that may reduce DENV replication while suppressed, constituting a mechanism for favoring flavivirus trafficking, conferring stability in the cell secretory pathway, and establishing a suitable pH environment for efficient virus secretion [37]. The multi-subunit enzymes, V-ATPases, are known to acidify various organelles such as lysosomes and components of the secretory pathway that facilitate processing and acid-dependent degradation of proteins during DENV infection [37]. An interaction of prM residues with V-ATPases is critical for viral egress. Additionally, ER-resident chaperones that are suggested to participate in the folding and assembly of the viral proteins are known to interact with the E protein [38]. The glycosylation of the E protein of the DENV at the residues 67 and 153/154 is important, as loss of either of the two E protein glycans can reduce the release of the virus in mammalian cells. The mature viral particles when released from the TGN enter the cytoplasm and further exocytose to the outer cell membrane [36,39,40]. As a final point, the cleaved-off pr and virions are released into the extracellular medium upon particle secretion [36]. A mature virion consists of one positive-sense single-stranded RNA encapsulated in multiple copies of C protein, surrounded by the lipid bilayer in which transmembrane viral proteins are inserted, forming a glycoprotein shell consisting of 180 copies of envelope and membrane proteins. Interestingly, the budding of empty virus particles of overexpressed prM and E proteins, lacking the capsid and viral RNA, is reported to be inferring with the budding of the immature virus particle, which is independent of RNA encapcidation [41]. Thus, the interactions between NC, PrM, and E protein need to be explored for a better understanding of the encapsidation and maturation of the DENV.

## 4. Pathogenesis of Dengue Virus inside the Host

The cells that first encounter the infection are skin resident macrophages and dendritic cells [11,42]. When these infected cells reach the lymph nodes, macrophages and monocytes are exposed to infection [11]. The DENV infection progresses to viremia due to the presence of the virus in drains and remote lymph nodes [43]. Infection of the DENV was previously observed in the spleen, kidneys, lungs, and liver through DCs, monocytes, and macrophages. These cells are among the most studied and considered to be the major sites of viral replication [11]. The viremia state can be detected as soon as 24–48 h before the onset of clinical symptoms and can last up to 10–12 days. If the mosquito feeds on blood during the viremic state of an individual, the mosquito gets infected and stays infected for life. The virus resides and multiplies within the mosquitos, and after an incubation period of 4–10 days, the mosquito transmits the virus to humans as well as passes it onto its offspring, thus increasing the pool of DENV vectors. Symptoms of the DENV infection include sudden onset of fever, body pain, headache, joint pain, rashes, and retro-orbital pain [44]. Although usually mild or asymptomatic, the DENV can progress into life-threatening serious conditions of hemorrhagic fever. The symptoms of mild hemorrhage include petechiae, purpura, ecchymoses, and epistaxis [11]. Mostly in children below 15 y of age and up to 2% of the total DENV cases experience progression of the infection to severe life-threatening conditions of Dengue Hemorrhagic Fever (DHF), which may cause liver damage and increase vascular permeability, thrombocytopenia, and the hemorrhagic manifestations that could affect the skin, nose, gum, and gastrointestinal tract [11,45,46]. Dengue Shock Syndrome (DSS), the most severe form of DHF, is characterized by a weak pulse and sudden drop in blood pressure, which is the result of the collapse of the vascular system owing to hypovolemia caused by vascular leakage [42]. The underlying mechanisms of development and progression of DENV infections to severe conditions are not well understood. Moreover, the variation in infectivity, severity, and the recovery of patients—i.e., a few people develop mild symptoms and recover quickly, while some develop severe life-threatening conditions and take longer to recover—is elusive.

## 5. Inflammation during Dengue Virus Infection

**Figure 3:**
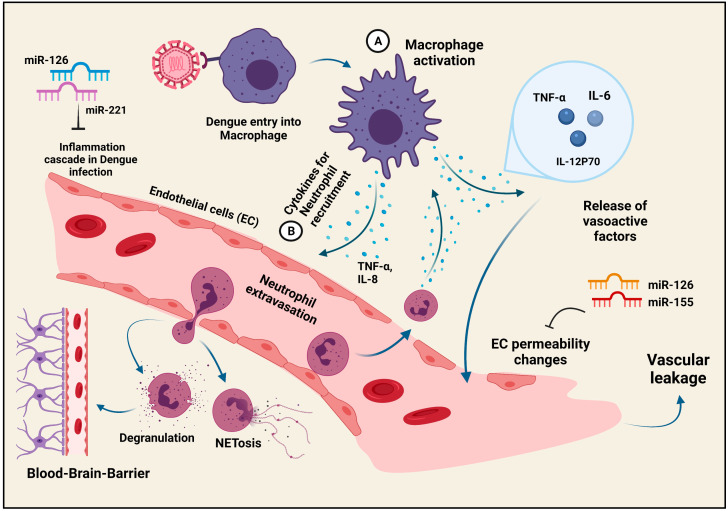
**Inflammatory responses during Dengue virus infection:** Dengue virus entry into the target macrophage cell triggers the inflammatory cascade. A. Macrophage activation leads to the increase in the vasoactive factors to induce permeability changes in the EC membrane ultimately leading to vascular leakage. B. Neutrophils, the major effectors in the inflammation process, are recruited by cytokines released from macrophages to migrate to the nearby tissues. Neutrophils under Dengue attack are capable of exhibiting NETosis, a phenomenon marked by the release of traps or NETs to capture the Dengue virus. Apart from NETosis, the neutrophils degranulate, an event that contributes to the penetration of the blood-brain-barrier to cause neuroinflammation. Certain miRNAs, miR-126, miR-155, and miR-221, which are potential rescuers of the EC permeability changes and inflammation, respectively, are being considered as therapeutic targets in order to reduce the damaging effects of inflammation in the Dengue patients.

Similar to any other viral infection, the cascade of inflammation marks the initial host response to the Dengue Virus attack (Figure 3). A localized inflammatory response represented by events such as neutrophil recruitment and extravasation, secretion of vasoactive factors for endothelial cell (EC) activation, as well as the generation of chemotactic agents to recruit the monocytes/macrophages to primary infection sites are all observed in the post-DENV attack, only to proceed onto a severe and prolonged inflammatory reaction that may be detrimental to the host [47]. The disturbing process, leading to a vascular leak syndrome, is the result of the changes in the EC permeability which are induced by the vasoactive chemokines and cytokines produced by the monocytes/macrophages or the dendritic cells (DCs) [48,49,50]. The macrophage or DC-produced cytokines, experiencing altered expression levels in the DENV infection, are TNF-α, IL-6, MIF, and certain metalloproteases [50,51]. Although the macrophages and the DCs may be the major inducers of EC permeability changes, the EC itself participates in the dysregulation of inflammation [52]. Studies have confirmed the role of Sphingosine kinase (SK1) in the TNF-α-mediated elicitation of inflammation via NFκB [53,54]. Netosis or NET (Neutrophil extracellular traps) formation by neutrophils is an interesting feature observed in patients suffering from Dengue Hemorrhagic Fever (DHF), with evidence of dire nuclei decondensation [55,56]. Other strategies employing microRNAs (miRNAs) to alleviate the severe inflammatory conditions in DENV patients are being explored. miRNAs are ~22-nucleotide-long RNA molecules that participate in the regulation of gene expression, targeting many genes via base complementarity [57]. Although the dysregulation of many miRNAs is observed in DENV-infected patients, very few were proven to be involved in DENV infection [58,59]. miR-30e*, which targets Iκβα, increases the IFNβ production to inhibit the DENV replication [60]. Similarly, other differentially regulated miRNAs, which are generally involved in EC permeability (miR-126, miR-155) and inflammation (miR-126, miR-221, etc.) and are elevated post-DENV infection, stand out as the prospective therapeutic agents against the DENV-induced vascular leak syndrome [61,62,63,64]. DENV, despite being a non-neurotropic virus, is associated with neuroinflammation and paralysis [65]. The effector functions of the microglial cells, neutrophils, natural killer cells, as well as the CD4+ and CD8+ T cells in the brain strongly indicate DENV-induced neurodegeneration as a result of excessive inflammation [66,67]. As far as the evasion of the blood-brain-barrier is considered, the participation of the Th-17 cells in neuro-evasion as well as neuroinflammation has been confirmed [68]. The interplay between the immune cells and DENV decides the outcome in the form of such manifestations, some of the interactions of which are described in the next section of the review.

## 6. Host Immune Cell Responses to Dengue Virus Infection

**Figure 4:**
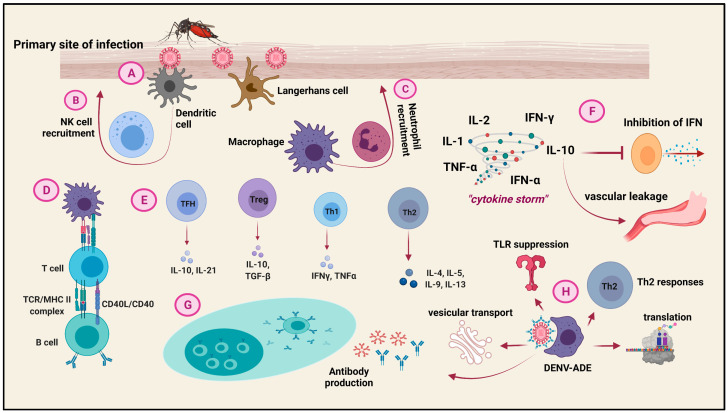
**Immune cells involved in the host responses to the Dengue virus:** After the mosquito bite injects the Dengue virus into the skin, A. The Langerhans cells and the Dendritic cells (DCs), the primary cells that encounter the Dengue virus, come into play. B. The DCs recruit the NK cells to the site, whereas, C. The macrophages recruit the neutrophils. D. Macrophages infected with DENV present the processed antigen to the T cell, which in turn interacts with the B cell to aid in B cell development. E. Different types of T cells are activated to elicit various cell-mediated immune responses against the virus. F. A gush of cytokines are secreted in an event called the “cytokine storm”, out of which the overproduction of IL-10 leads to the inhibition of IFN responses as well as vascular leakage. G. The humoral immunity, represented by the B-cell-mediated antibody production from the plasma cells, as a natural immune response to any pathogen, induces ADE in the DENV infection. H. The ADE of the DENV entry into the macrophage via phagocytosis leads to an altered transcriptome of the cell, ultimately leading to TLR suppression, enhanced Th2 responses, along with the dysregulation of vesicular transport and the host translation process.

Both the innate and the adaptive immune systems of the host network cooperatively rebel against the DENV attack (Figure 4). The Langerhans cells (LCs), DCs, and the dermal cells at the site of the mosquito bite are the first encounterers of DENV [69]. The distribution of DCs in the tissue is such that they do not miss an encounter with the virus [70]. They along with the macrophages are recruited to the site of the DENV entry via signals from the chemoattractants. The migration of the antigen-presenting cells independently contributes to inflammation as well as the host adaptive responses [71]. Signals from DCs, such as TNF-α, recruit NK cells, which are crucial in restricting DENV replication and pathogenesis during the early stages of infection by IFN production [72]. Neutrophils are also recruited to the primary site of infection by the tissue-resident macrophages through the secretion of IL-8, TNF-α, and IFN-β. Antiviral factors such as TNF-α and defensins are generated by neutrophils [55]. Moreover, the degree of complement activation by the DENV-NS1 protein is greater in DHF patients than in mild/intermediate Dengue Fever (DF) [73]. The effector functions of these immune cells are the result of certain innate immune pathways and their interactions with each other. The dsRNA intermediates of DENV are recognized by TLR-3, which through the activation of TRIFF lead to the phosphorylation and activation of the interferon regulatory factors IRF-3 and IRF-7 [74]. The TLR pathogen recognition pathway leads to the production of class I IFNs that restrict DENV replication by successively activating the JAK/STAT pathway [75]. Other receptors participating in the IFN-mediated combat against DENV are RIG-1 and MDA5 [76]. By the time, the innate immune system attempts to restrict the virus; the adaptive immune system prepares for an advanced attack for virus clearance. The B cells bind to the DENV antigens to elicit a primary IgM response, followed by an elaborate DENV-serotype-specific-IgG response. The secondary infection is marked by a reduced IgM but an enhanced IgG response [77]. The antibodies basically target the NS1, E, and prM proteins of the DENV [78]. The involvement of B cells also indirectly points to the involvement of T cells in the DENV infection response. While CD4+ T cells mainly target the capsid along with the NS2A/B, 3, 5 and E proteins, the CD8+ T cells target the capsid along with the NS3, 4A/B, 5 proteins [79]. These responses prompt the CD8+ T cytotoxic cells to secrete perforins and granzymes and the CD4+ T cells to involve in Th1 (secreting IFN-γ and TNF-α) effector functions. Moreover, the other T cell subsets such as the Treg cells act to regulate inflammation via TGF-β and IL-10, and the Follicular T cells function to aid the B cells into generating highly specific antibodies with sufficient affinity [55,80]. Although the entire immune system works together to put up a prodigious fight against DENV, some of these responses may in turn cause host damage, through an event known as the “cytokine storm”.

## 7. Cytokine Storm during Dengue Infection

Dengue Hemorrhagic Fever (DHF) is characterized by extreme vascular permeability leading vascular leakage. DHF may ultimately lead to Dengue Shock Syndrome (DSS), which is characterized by decreased peripheral perfusion leading to tissue damage and the failure of multiple organs [2]. The event of the “cytokine storm”, referring to heightened production of cytokines (Figure 4), majorly, IL-1, IL-2, IL-10, CXCL-10, CCL-2, VEGF, TNF-α, IFN-α, and IFN-γ, is observed in both conditions of DHF and DSS, which is not evident in mild/intermediate DF [81,82,83,84]. The event of the cytokine storm is a result of the imbalance between the Th1 and Th2 cytokine responses. Overproduction of Th2 cytokines, such as IL-10, ends up overpowering the IFN responses leading to augmented DENV viral load, which is often observed in DHF patients [85]. Increased IL-10 levels may also contribute to plasma leakage [86]. Moreover, cytotoxic factors released upon the activation of CD4+ Th cells trigger the macrophages to produce reactive nitrogen species that consecutively encourage the Th2 responses, ultimately instigating permeability changes and plasma leakage [2]. Although the antibodies produced during the primary DENV infection are extremely serotype-specific, during infection with a heterologous serotype, low-avidity memory cells may crosslink with the antigen to elicit a cytokine storm and result in similar complications [87].

## 8. Intrinsic Antibody Dependent Enhancement (ADE) during Dengue Infection

While the general Dengue infection follows the receptor-mediated endocytic pathway for viral entry into the target cells, entry of the antibody-opsonized DENV in the ADE condition follows a phagocytosis pathway into the macrophages or the DCs by means of FcγR crosslinking [88]. This calls for a type I IFN response. However, the DENV escapes the IFN responses by binding to the inhibitory receptor, LILRB1, or the leukocyte immunoglobulin-like receptor B1 to inhibit the expression of the Interferon Stimulatory Genes (ISGs) [89]. This enhances the antibody-dependent DENV infection in cells. The implications of the Dengue-ADE infection include the enhancement of differentially-expressed genes (DEGs), some of which directly communicate with the DENV RNA [90]. ADE of the Dengue infection also causes an altered transcriptome in the monocytes, increasing gene expression associated with the processing of mRNA and transport of vesicles. DEGs associated with the host translation process are also increased as opposed to the DEG downregulation in DF infections. The DENV adapts this strategy to divert the host translation process towards the translation of the viral proteins [91,92]. Another opposing characteristic of Dengue-ADE is the suppression of TLR expression during secondary infection with a heterologous DENV-serotype. This is achieved by the upregulation of TANK and SARM, the negative regulators of the TLR pathway [93,94]. Downregulation of TLR-3, -4, and -7 disrupts IRF-1 and -3 expression, leading to aberrant TLR signaling via the non-NFκB-mediated pathway [93,94]. DENV replication and translation is also increased due to the enhanced potential for membrane fusion in primary macrophages that succumb to the antibody-dependent DENV infection [95,96]. In order to dampen the host immune responses, ADE of the DENV causes heightened IL-10 production. This along with the overproduction of IL-6 establishes a shift towards the Th2 responses [85,94]. The overproduction of these two cytokines leads to the inhibition of the JAK/STAT pathway via SOCS3 overexpression, to inhibit IFN-γ production and, therefore, the Th1 responses. The synthesis of Nitric Oxide (NO), which strongly annuls DENV replication, is abrogated itself consequentially [92,94]. The anti-inflammatory milieu and the Th2 dominance promote the proliferation of the B cells to encourage more antibody production. This exacerbates the already worsened situation to generate antibodies to facilitate the antibody-dependent DENV entry into the host cells [92].

## 9. Dengue Virus Induced Cell Death

Cell death during viral infections is crucial in the replication and spread of viral pathogenesis. During the initial stages of infection, when the virus has not yet overpowered the cell machinery, the cellular processes try to induce cell death as a mechanism to inhibit the replication and spread of the virus to the neighboring uninfected cells. Later, when the virus establishes its infection, it attempts to modify the cellular induced death processes for its own benefit. The DENV, similar to other viruses, modifies the induced cell death processes of apoptosis and autophagy to establish a productive infection in the host.

### 9.1. Regulation of Apoptosis during Dengue Virus Infection

**Figure 5:**
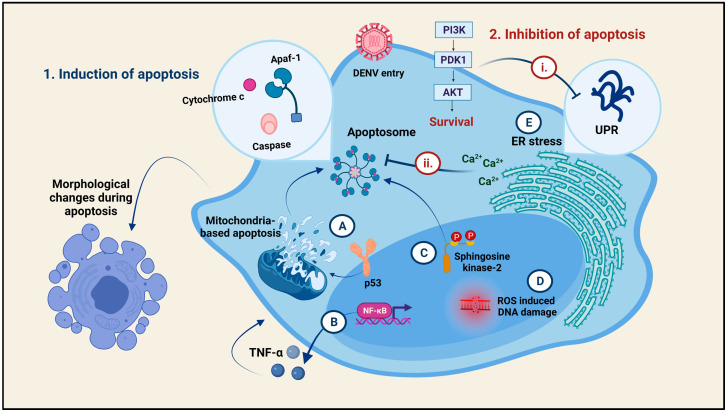
**Regulation of cell death via apoptosis during Dengue virus infection:** The Dengue virus is capable of 1. Inducing apoptosis as well as 2. Inhibiting apoptosis in target cells, depending upon its requirement. The different mechanisms of apoptosis induction are: A. Induction of the mitochondria-based apoptosis by the pro-survival protein, p53, to assemble the apoptosome for Caspase-3, 9 activation. B. The NFκB-TNF-α mediated induction of cell apoptosis. C. The Sphingosine-kinase-2 mediated induction of apoptosome formation. D. ROS-induced DNA damage that ultimately leads to the death of the cell. E. Induction of ER stress to activate the UPR responses in the ER of the cell. All these mechanisms result in morphological changes in the cell indicative of apoptosis-mediated cell death. The DENV also allows the survival of the cell so as to continue parasitizing it. The anti-apoptotic mechanisms include: i. Increased Akt phosphorylation to inhibit the ER-stress-induced UPR responses in the cell and ii. Enrichment of cytoplasmic calcium ions to inhibit the apoptosome formation.

Dengue virus is capable of having reciprocating effects on apoptosis regulation in cells (Figure 5). All the four DENV serotypes potentially induce cellular apoptosis via the direct involvement of the cytotoxic M-proteins [97]. Cell-death-inducing proteins, such as RIPK2 and Daxx, are utilized during capsid-induced apoptosis [98,99]. DENV-1 induces apoptosis by the activation of the NFκB transcription factor and induction of ER stress [100]. The prM and the C proteins of DENV-1 trigger the mitochondria and p53-based apoptosis in the HUH-7 cells [100]. On the other hand, DENV-serotype-2 participates in the pro- as well as the anti-apoptosis process. The pro-apoptotic functions of DENV-2 involve the activation of the Caspase-3 through the activation of XAF-1 (an IFN-inducing, XIAP-associated factor) to trigger apoptosis within 36 h of infection [101]. The intrinsic apoptotic pathway characterized by morphological changes, such as blistering of the plasma membrane, contracting of the cytoplasm, and swelling of the mitochondria, followed by the activation of caspase-9 and caspase-3, resulting in cleavage and apoptosis, is also well reported in the DENV-2 infection [102,103,104]. Moreover, p53 phosphorylation and a reduction in the Bcl-2 anti-apoptotic factor are indicative of the involvement of the intrinsic apoptotic pathway. Similar to DENV-1, DENV-2 is also involved in the related pathway. 

Stress response pathways such as the Unfolded Protein Response (UPR) and the Noxa/PUMA lead to apoptosis induction [105]. Moreover, DENV-2 triggers caspase activation via NFκB-mediated TNF-α expression [106]. Since oxidative stress is a positive transducer of apoptosis, DENV-2-infected PBMCs showed a positive relationship between oxidative stress, DNA damage, and apoptosis [107,108]. Having said so, NO, a secondary messenger, acts as a mediator of caspase-dependent apoptosis [109]. Moreover, sphingosine kinase-2 or SPHK2 has a significance in inducing the intrinsic apoptotic pathway in cells via the activation of caspase-3 and -9 [110]. The anti-apoptotic functions of DENV-2 are mediated by the inhibition of XBP-1 and increased Akt phosphorylation, so as to suppress the UPR stress response sequentially, in order to prohibit early apoptosis and extend the survival of the DENV by keeping the cell alive [111,112]. One of the other factors involved in apoptosis inhibition is the enrichment of calcium in the cell cytoplasm to protect the cell from apoptosis via mitochondrial damage [97,113].

### 9.2. Regulation of Autophagy during Dengue Virus Infection

**Figure 6:**
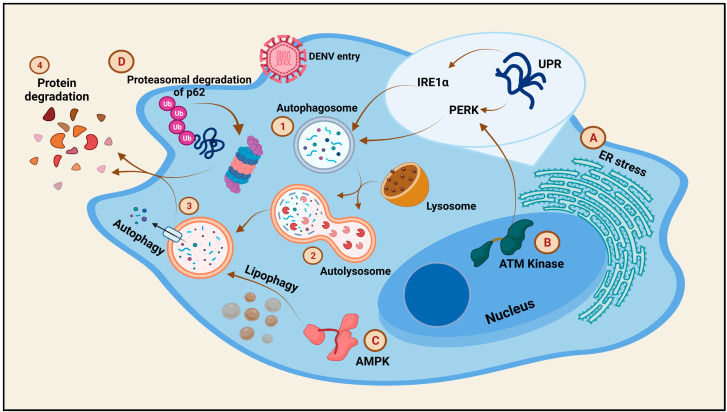
**Regulation of cell death via autophagy during Dengue virus infection:** The entry of Dengue virus induces autophagy in the infected cell. The mechanisms of autophagy post-DENV infection are: A. Induction of ER stress to trigger the UPR responses, out of which 2 pathways, involving IRE1α and PERK, respectively, lead to the formation of autophagosomes in the cell. B. Autophagosome formation is also triggered by the involvement of ATM kinase to trigger the PERK pathway for autophagosome formation. C. AMPK, a kinase involved in lipophagy, is also exploited by the DENV to modulate the metabolic status of the cell. D. Apart from the common steps of autophagy, the DENV employs the proteasomal degradation of p62 to enhance its replication. The autophagosome in step 1 fuses with the lysosome to form the 2. Autolysosome structures which ultimately lead to 3. Autophagy and 4. Protein degradation.

The events of triggered autophagy have been observed in DENV-infected cells. While DENV-induced autophagy in monocytes leads to a drop in the virus proliferation, the same in HUH-7 cells leads to a productive infection [114,115]. Autophagy triggered by the DENV infection is primarily mediated by three pathways, namely, the ER stress, AMPK, and the Ataxia telangiectasia mutated (ATM) kinase pathways (Figure 6).

As mentioned above, the DENV induces ER stress. During this, a large number of unfolded proteins are accumulated in the ER which in turn induce responses from three UPR pathways: IRE1α, PERK, and ATF6. IRE1α triggers mRNA splicing of the XBP-1 transcription factor, in response to the induction of ER stress. XBP-1, with assistance from Beclin-1. increases the LC3 expression, leading to the formation of autophagosomes [116,117]. Moreover, IRE1α activates the autophagic pathway via the participation of the c-Jun-N-terminal kinase. PERK is also an activator of autophagy in DENV-infected cells, via the participation of the eukaryotic translation initiation factor-2α (eIF2α), although the role of ATF6 in autophagy has yet to be reported [118,119]. AMPK is a regulator of the host lipid metabolism capable of inhibiting mTORC1 to induce lipophagy in cells. DENV exploits the AMPK pathway of lipophagy induction to generate sufficient energy from the lipid metabolism for efficient DENV replication [117]. The ATM kinase pathway, which is involved in the regulation of oxidative stress in cells, is induced to generate reactive oxygen species (ROS) via the PERK pathway, subsequently leading to autophagy in DENV-infected cells [118]. Contradictorily, the p62 autophagic receptor production is reduced by the DENV by targeting the protein for proteasomal degradation, a phenomenon important for DENV replication [120]. Therefore, the DENV is capable of manipulating the process of autophagy in the cells depending on the stage of infection and the host cell responses.

## 10. Antivirals against Dengue Infection

With no commercial antivirals available for public use against the Dengue virus and severe Dengue infections leading to conditions such DHF and DSS, the discovery of potential anti-DENV agents is still a pressing issue. Drugs used to treat other conditions, such as the anti-pyretic drug Acetaminophen and the anti-hepatitic Sofosbuvir, were also shown to reduce the Dengue infection symptoms to some extent [121,122]. The vaccine Dengvaxia which was given the license for use in about 20 countries recently began to show some complications in seronegative patients [123]. Two other vaccines undergoing clinical trials are TAK-003 (developed by Takeda Inc. Japan) and TV003 (developed by the National Institutes of Health, NY, USA), out of which the TV003 vaccine was proposed to display neutralizing effects against multiple DENV serotypes [124,125]. Moreover, a Dengue Virus Serotype 1 mRNA-LNP vaccine was reported to show promising, protective immunological responses during in vivo studies [126]. A siRNA targeting the DENV-NS4B and -NS5 were found to silence the disease progression of all the DENV-serotypes (1–4) successfully [127]. Since Dengue is capable of modulating the host machinery and cellular resistances to increase the disease severity, studies revealing the anti-DENV properties of natural compounds are appreciated, due to their increased bioactivity and stability compared to synthetic drugs, along with an extended receptor range owing to the process of evolution. In vitro and in vivo studies with Geraniin (an ellagitannin compound obtained from multiple plant species) was shown to restrict the virus-cell receptor process to inhibit the early DENV replication stages, leading to decreased viremia [128]. Another phenolic compound, Quercetin (a flavanol), potentially downregulates TNF-α in an attempt to control the excessive inflammation [129]. Phorbol ester (PMA) is also an inhibitor of the TNF-α levels post-DENV infection [130,131]. Although there are many such phenolic compounds, such as, Fisetin, Naringin, Catechin, and Delphinidin, that showed inhibitory effects on the DENV in pre- and post-treatments, their mechanisms of inhibition have yet to be clarified [132,133,134,135]. Resveratrol, a phytoalexin antiviral to other Flaviviruses, was also shown to attack the DENV genome directly [136]. One of the phenolic compounds that has its inhibition mechanisms well elucidated is Nordihydroguaiaretic acid, which can reduce DENV-NS1 protein production and prevent the accurate assembly of the DENV virions [137]. Curcumin and Salidroside affect the structure of the DENV envelope and the expression of IRF3, respectively [138,139]. Policresulen inhibits the NS2B/NS3 protease of the DENV to restrict viral replication in BHK-21 cells [140]. Honokiol is another potent inhibitor of the early DENV pathogenesis steps in BHK cells by the downregulation of the NS1 and NS3 proteins as well as the RNA intermediates of the DENV [135,141]. Diterpenes and derivatives from *Curcuma longa* and *Basilicum polystachyon* were also found to be effective against the DENV [142,143]. Luteolin was reported to be an uncompetitive inhibitor of the host proprotein convertase furin, which is an endoprotease significant in DENV maturation in the trans-Golgi compartment [144]. Apart from the specific bioactive compounds, many of the medicinal plants, such as *Acacia catechu*, *Acorus calamus*, *Allium sativum*, *Azadarachta indica*, *Cissampelos pareira*, *Cymbopogon citratus*, *Ficus septica*, *Kaempferia parviflora*, *Myristica fatua*, *Pavetta tomentosa*, and many more compiled by Lim et al., 2021, showed anti-DENV effects and await research pertaining to their mechanism of action for the development of efficient therapeutics against the DENV [145].

## 11. Conclusions

Dengue virus has been elaborately studied over the years to understand the pathogenesis in order to treat the associated conditions of DHF and DSS. In this review, we tried to compile the areas of DENV research that were thus far explored, as well as those that demand more attention. An understanding of the coordination of the cellular responses along with the manipulation of the resistance offered by the host is crucial in the discovery of prospective DENV antivirals that prove effective against all the DENV serotypes. Moreover, the ability of numerous medicinal plants in reducing the effects of the DENV suggests that an expansion of research in this field is indispensable. Together, this review attempts to shed light on the various aspects of the DENV lifecycle and pathogenesis, opening doors to new ideas and concepts towards the extenuation of the virus and the attenuation of host damage.

Although a lot is known about the DENV, it seems to be a tip of the iceberg, as multiple roles of viral proteins, and the governance of the viral genome conformation in DENV multiplication, are being studied that would prove their eminence.
